# TMSEEG: A MATLAB-Based Graphical User Interface for Processing Electrophysiological Signals during Transcranial Magnetic Stimulation

**DOI:** 10.3389/fncir.2016.00078

**Published:** 2016-10-07

**Authors:** Sravya Atluri, Matthew Frehlich, Ye Mei, Luis Garcia Dominguez, Nigel C. Rogasch, Willy Wong, Zafiris J. Daskalakis, Faranak Farzan

**Affiliations:** ^1^Temerty Centre for Therapeutic Brain Intervention, Centre for Addiction and Mental HealthToronto, ON, Canada; ^2^Institute of Biomaterials and Biomedical Engineering, University of TorontoToronto, ON, Canada; ^3^Department of Electrical and Computer Engineering, University of TorontoToronto, ON, Canada; ^4^Brain and Mental Health Laboratory, School of Psychological Sciences and Monash Biomedical Imaging, Monash Institute of Cognitive and Clinical Neuroscience, Monash UniversityMelbourne, VIC, Australia; ^5^Department of Psychiatry, University of TorontoToronto, ON, Canada

**Keywords:** transcranial magnetic stimulation, electroencephalography, artifact correction, MATLAB toolbox, signal processing, independent component analysis, standardized workflow, brain mapping

## Abstract

Concurrent recording of electroencephalography (EEG) during transcranial magnetic stimulation (TMS) is an emerging and powerful tool for studying brain health and function. Despite a growing interest in adaptation of TMS-EEG across neuroscience disciplines, its widespread utility is limited by signal processing challenges. These challenges arise due to the nature of TMS and the sensitivity of EEG to artifacts that often mask TMS-evoked potentials (TEP)s. With an increase in the complexity of data processing methods and a growing interest in multi-site data integration, analysis of TMS-EEG data requires the development of a standardized method to recover TEPs from various sources of artifacts. This article introduces TMSEEG, an open-source MATLAB application comprised of multiple algorithms organized to facilitate a step-by-step procedure for TMS-EEG signal processing. Using a modular design and interactive graphical user interface (GUI), this toolbox aims to streamline TMS-EEG signal processing for both novice and experienced users. Specifically, TMSEEG provides: (i) targeted removal of TMS-induced and general EEG artifacts; (ii) a step-by-step modular workflow with flexibility to modify existing algorithms and add customized algorithms; (iii) a comprehensive display and quantification of artifacts; (iv) quality control check points with visual feedback of TEPs throughout the data processing workflow; and (v) capability to label and store a database of artifacts. In addition to these features, the software architecture of TMSEEG ensures minimal user effort in initial setup and configuration of parameters for each processing step. This is partly accomplished through a close integration with EEGLAB, a widely used open-source toolbox for EEG signal processing. In this article, we introduce TMSEEG, validate its features and demonstrate its application in extracting TEPs across several single- and multi-pulse TMS protocols. As the first open-source GUI-based pipeline for TMS-EEG signal processing, this toolbox intends to promote the widespread utility and standardization of an emerging technology in brain research.

## Introduction

Non-invasive brain stimulation is an emerging approach for the assessment and improvement of brain health. Transcranial magnetic stimulation (TMS) is one such approach with a growing range of applications across multiple disciplines of neuroscience. In TMS, time-varying currents in an induction coil held near the scalp can induce magnetic fields. These time-varying magnetic fields then generate electrical currents in the targeted brain regions (Ilmoniemi and Kičić, 2010). By varying stimulation parameters, a number of TMS paradigms have been developed to assess and modulate brain function (Hallett, 2000). For example, single- or paired-pulse TMS paradigms have been designed to assess the functional integrity of neural circuits (Valls-Solé et al., 1992; Pascual-Leone et al., 1998; Walsh and Cowey, 2000) and repetitive TMS (rTMS) paradigms can be used to assess brain plasticity and induce transient modulation of brain function (Thut and Pascual-Leone, 2010). To quantify the impact of TMS on brain tissue, stimulation is traditionally applied over the primary motor cortex. Cortical reactivity is then quantified by characteristics of motor-evoked potentials (MEPs) captured through peripheral electromyography (EMG) recordings. To directly quantify the impact of TMS on brain tissue, cortical reactivity can be assessed through electroencephalography (EEG), a non-invasive method for measuring electrical brain activity through scalp measurements. In recent years, the combination of TMS with EEG (TMS-EEG) has allowed the direct evaluation of TMS effects through TMS-evoked potentials (TEP)s. This combination has been instrumental in expanding the utility of TMS beyond the motor system (Ilmoniemi and Kičić, 2010; Miniussi and Thut, 2010; Daskalakis et al., 2012).

**Figure 1:**
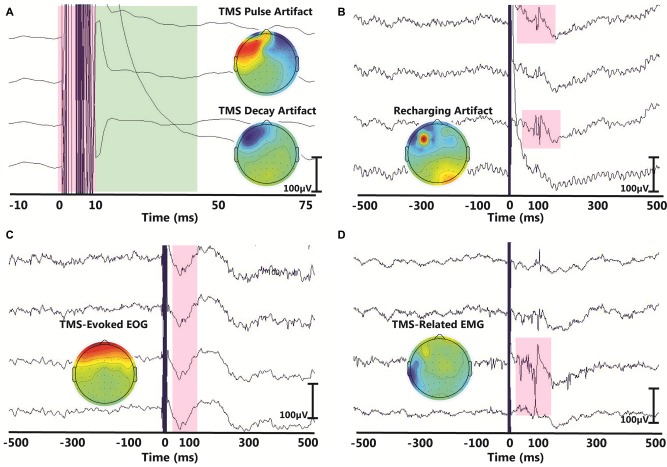
**Examples of common artifacts in transcranial magnetic stimulation-electroencephalography (TMS-EEG) data.** In each panel, the *x*-axis represents time in the millisecond range and *y*-axis represents the amplitude of the EEG signal in the microvolt range. Topographical maps in each panel illustrate the scalp projection of each corresponding artifact. The displayed artifacts are: **(A)** the large-amplitude TMS pulse artifact (red) with the associated TMS decay artifact (green), **(B)** recharging artifact, **(C)** TMS-evoked eye blink artifact and **(D)** TMS-related muscle artifact.

Despite a growing interest in TMS-EEG methodology, challenges inherent to TMS-EEG signal processing and the lack of standardization have impeded its wider adoption. TMS can induce several types of artifacts in EEG data, which can easily mask small-amplitude EEG signals (Figure 1). As such, proper analysis and interpretation of TMS-EEG data must address the challenge of extracting artifacts from TMS-EEG recordings without distorting the underlying TEPs. To date, several studies have documented physiological and non-physiological artifacts that can mask or distort TEPs (Ilmoniemi and Kičić, 2010; Miniussi and Thut, 2010; Ilmoniemi et al., 2015) and impact TMS-EEG data interpretation (Rogasch et al., 2014). Among the identified artifacts, there is an early large-amplitude TMS electromagnetic pulse artifact (Figure 1A) that can be caused or prolonged by amplifier saturation (Ives et al., 2006; Ilmoniemi and Kičić, 2010; Vernet and Thut, 2014; Ilmoniemi et al., 2015). Accompanying the TMS pulse artifact is often a decay artifact (Figure 1A), which results from the slow decay of TMS-induced charge accumulation at interfaces with capacitive properties such as the electrode-electrolyte-skin interface (Veniero et al., 2009). In addition, TMS-EEG is often contaminated by artifacts that may or may not be time-locked to the TMS pulse such as the recharging artifact (Figure 1B), eye movements or electrooculographic (EOG) artifacts (Figure 1C; Lins et al., 1993; Jung et al., 2000; Lyzhko et al., 2015), muscle artifacts (EMG; Figure 1D; Mäki and Ilmoniemi, 2011; Mutanen et al., 2013), electrode movement artifacts (Sekiguchi et al., 2011), and auditory-evoked potentials (AEPs; Nikouline et al., 1999). AEPs are neural responses induced by the clicking sound of each TMS pulse through both air and bone conduction when a TMS coil is held on the scalp. AEP components in EEG are time-locked to the TMS pulse and can span a time window of roughly 200 ms masking TEPs (Starck et al., 1996; Rogasch et al., 2014). To this end, several algorithms have been proposed to recover brain response from TMS-related artifacts (Mäki and Ilmoniemi, 2011; Mutanen et al., 2013; Ilmoniemi et al., 2015; Lyzhko et al., 2015). While some early approaches simply remove EEG data segments contaminated with noise, many of the more recent correction methods use blind source separation techniques to recover the brain signal. In blind source separation, TMS-EEG signals are decomposed into independent or orthogonal components via independent component analysis (ICA) or principal component analysis (PCA). After decomposition, noise components are removed and the remaining components are used to reconstruct the signal. The efficacy of blind source separation in removing TMS-related artifacts has been documented previously and will not be discussed here (e.g., Litvak et al., 2007; Mäki and Ilmoniemi, 2011; Rogasch et al., 2014; Lyzhko et al., 2015). Moreover, an in-depth discussion of TMS-EEG methodology, TMS-EEG applications, TMS-related artifacts, and the rationale behind the methodological choices in this article are provided in a recent review article (Farzan et al., 2016).

Despite the multitude of studies on the processing of TMS-EEG data, there is yet to be a comprehensive and standardized toolbox for TMS-EEG processing. A strong motivation for a standardized workflow is to ensure data consistency, enable data comparisons across subjects, studies and investigators, and promote the application of TMS-EEG in various fields of research. Thus we propose TMSEEG, a MATLAB (The Mathworks, Inc., Natick, MA, USA) application dedicated for TMS-EEG signal processing. When implemented with a modular graphical user interface (GUI), TMSEEG provides an interactive and compact platform to process data from several TMS-EEG paradigms. The GUI is designed to guide novice users and support the integration of alternative or additional processing modules for more advanced users. The purpose of this article is to describe the toolbox and its underlying software architecture, and justify the choices made in the design of the framework.

To simplify and standardize TMS-EEG signal processing, the toolbox integrates five main novel approaches:

1. Integration of algorithms to enable the removal of TMS-specific and general artifacts from EEG data;
2. A streamlined yet modular and modifiable TMS-EEG data processing workflow;
3. A comprehensive multi-panel and multi-dimensional display of TMS-EEG artifacts;
4. Online update and visualization of TEP waveforms throughout the processing steps; and
5. The ability to label and store information marked as artifacts in a database.

## Materials and Methods

TMSEEG is developed in MATLAB (R2013a) and built upon the widely used data processing tool EEGLAB (v.12.0.2.6b; Delorme and Makeig, 2004). In this section, we briefly describe the steps of the toolbox workflow. As this is not intended to be a user manual, we invite the reader to visit http://www.tmseeg.com/ for further information and a detailed tutorial on the functional aspects of TMSEEG.

### Graphical User Interface

**Figure 2:**
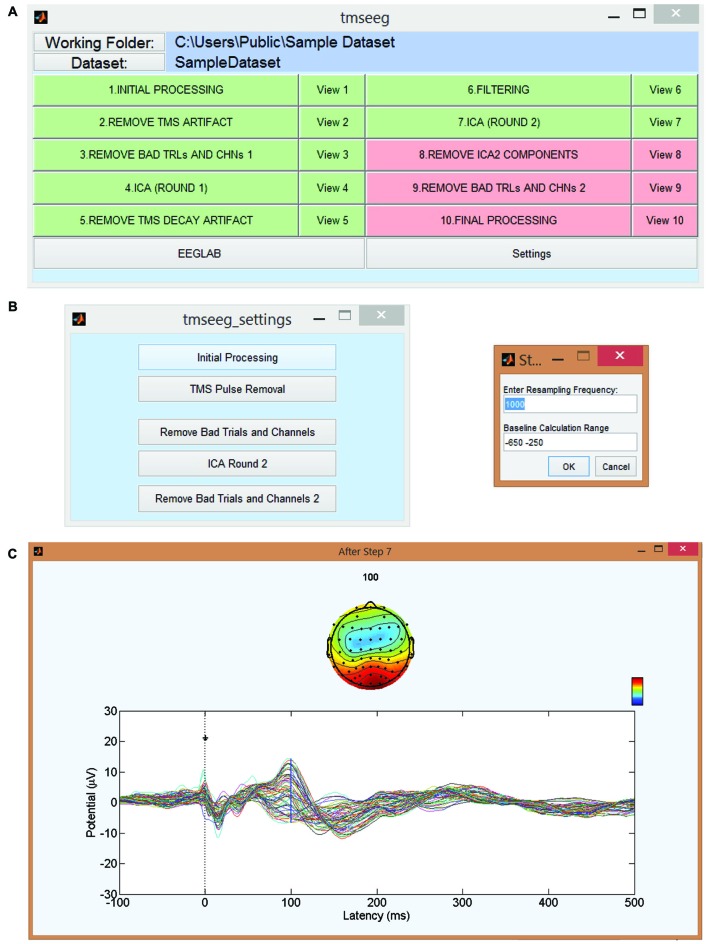
**TMSEEG main graphical user interface (GUI). (A)** TMSEEG main GUI window comprised of a button for TMS-EEG file selection, 10 data processing buttons, 10 data View buttons, and two buttons for access to the Settings menu and EEGLAB toolbox. Data processing buttons are coded in red if a step is yet to be processed and changed to green at the completion of a step. **(B)** Users can change the default settings of any processing step in the corresponding Settings window. **(C)** Selecting the “View #” button next to a processing step will display the average butterfly plot illustrating TMS-evoked potentials (TEP)s at the completion of that step for a quality control check. *X*-axes represent time in milliseconds relative to presentation of TMS pulse and *y*-axes represent the amplitude of the EEG signal in μV.

TMSEEG can be accessed through the apps toolbar in MATLAB or by running the *tmseeg_main* function. Both of these approaches initialize the Main GUI window depicted in Figure 2. This parent GUI allows the user to: (i) specify the working folder; (ii) load the unprocessed data; (iii) run several processing steps (to be described below); (iv) display the average TEP waveform at the completion of each processing step (“View Step” Button); (v) specify the settings for each processing step; and (vi) access EEGLAB. The data processing buttons in the parent GUI appear in green if the step has been completed successfully or in red if a step has not been completed.

### Integration With EEGLAB

TMSEEG is built upon EEGLAB to allow the sharing of functionality between the two toolboxes. EEGLAB functions are used across the processing steps of TMSEEG for loading, resampling, channel editing, ICA, channel interpolation, and re-referencing. In addition to integrating these functions, TMSEEG also provides several unique functions to process TMS-EEG data, including artifact quantification and an interactive interface for artifact rejection. These features will be highlighted in the following sections. Advanced users may modify TMSEEG to include customized algorithms within the workflow.

### Data Structure

TMSEEG uses the EEGLAB format (i.e., data structure) to save data, meta-parameters and analysis parameters. Artifact information, including tagged (marked for deletion) trials and ICA components are saved in a separate MATLAB file for later retrieval and modification. An EEGLAB compatible dataset file (*.SET file format) is saved at the completion of each processing step, allowing users to easily revert to an earlier step for re-processing if necessary. This dataset file is automatically saved using standardized naming conventions in the specified working folder to track and assess the progress of the data processing workflow.

### Step-by-Step Procedure

The TMSEEG toolbox incorporates 10 steps of data processing to recover brain signals contaminated by different artifacts. To conceptualize the general framework of TMSEEG, artifacts targeted by the toolbox can be broadly divided into two classes: consistent artifacts and variable artifacts. Consistent artifacts have highly reproducible temporal, spectral, or spatial distributions that can be extracted using filtering or component-based methods, while preserving the underlying data. These include the TMS decay, electrical noise, EMG, EOG, EKG or AEP artifacts. Variable artifacts, however, have irregular characteristics and may not be easily identified with component-based methods. These include the large-amplitude TMS pulse artifact and other sporadic artifacts that are not well-defined in the spatial, temporal, or frequency domain. Removing data segments with variable artifacts can improve the performance of component-based methods and allow the separation of the remaining data into independent neural and consistent-type artifact components. The TMSEEG toolbox is organized to follow the principle that artifacts larger in amplitude or variance are removed earlier in the pipeline in the following order:

1. **Large-amplitude TMS pulse artifact (Step 2)**: This is an early, short-lived electromagnetic artifact of the TMS pulse which has a large bandwidth and no distinct spatiotemporal characteristics. This artifact is not easily detected by ICA and its large amplitude may limit the application of some artifact rejection algorithms to the data. In TMSEEG, the time segment contaminated with this artifact (e.g., <10 ms) is removed early in the workflow. The duration of this time window may vary as a function of the recording system, the site of stimulation (e.g., depending on the site of stimulation, TMS may induce a large-amplitude TMS-evoked EMG artifact which may overlap with or prolong the large-amplitude pulse artifact), coil orientation, TMS protocol, or various other factors. In Step 2, users have the capability to identify the time window for their respective dataset.
2. **Sporadic artifacts (Steps 3 and 9)**: Data must be thoroughly inspected to identify and remove trials/channels with sporadic artifacts resulting from poor electrode contact, movement in electrode wires, or subject’s movement. The presence of sporadic noise can hinder the performance of ICA, therefore data segments with these artifacts are removed in Step 3 prior to the first round of ICA. This inspection is performed again at the second last step (Step 9) of the pipeline to remove any remaining artifacts.
3. **TMS decay artifact (Step 4–5)**: With the early TMS pulse artifact removed, a first round of ICA is applied to identify the TMS decay artifact. Since the shape of the TMS decay artifact waveform is defined by a characteristic exponential decay, it can be identified, attenuated or removed using ICA.
4. **Signals outside the bandwidth of the EEG signal (Step 6)**: Zero-phase filtering is used to exclude signals outside the bandwidth of interest. The filtering step is performed after extracting the large-amplitude TMS artifact from the data (in Step 2) to prevent ringing artifacts. It also precedes the second round of ICA (Steps 7–8) to prevent the loss of ICA components to sources outside the bandwidth of interest.
5. **TMS-induced and general EEG artifacts (Steps 7–8)**: A second round of ICA is applied to extract TMS-evoked artifacts with recurrent spatiotemporal or spectral characteristics such as eye blinks or eye movements (EOG), muscle twitches (EMG) and AEPs. This step also aims to remove general EEG artifact components such as random eye blinks or eye movement (EOG), random muscle movement (EMG), electrocardiographic (EKG) or cardiac signals, and electrode movement.

Throughout the TMSEEG pipeline, the user has the capability to monitor the effect of deleting any single trial, channel or component on TEP quality. Although the presented pipeline can be suitable for most TMS-EEG data, the strategy for data cleaning may also vary depending on the experimental protocol (e.g., site of TMS administration, TMS-EEG paradigm). In TMSEEG, users have the flexibility to modify the software implementation and add customized data processing algorithms to the pipeline.

**Figure 3:**
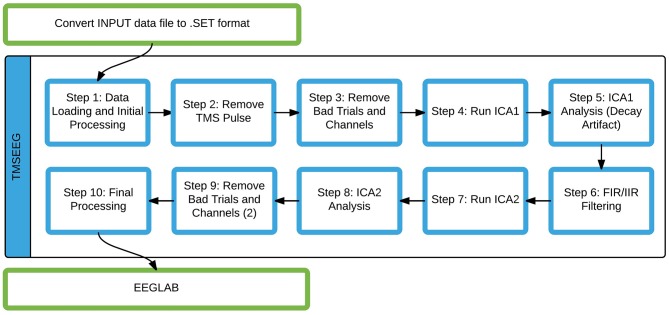
**The TMSEEG data processing workflow.** Steps highlighted by blue boxes are modular functions within the TMSEEG processing workflow. The program takes an input file in .SET format (conversion done in EEGLAB), creating intermediate datasets after each step for easy reprocessing.

The current sequential data processing workflow implemented in TMSEEG is illustrated in Figure 3. In the following sections, each step of the pipeline will be described in detail to address three key points: main function, novel features, and a description of the GUI. The step-by-step data processing workflow is illustrated using sample TMS-EEG data collected during the application of single-pulse TMS to the left primary motor cortex at rest.

#### Step 1. Initial Processing

The initial processing step includes: (i) locating the base data file in .SET format; (ii) downsampling the EEG data (optional); (iii) importing EEG channel coordinates; (iv) deleting user-defined channels such as auxiliary or disconnected channels (optional); (v) epoching; and (vi) baseline removal. Initialization parameters for these tasks can be specified in the settings tab located in the Main GUI of TMSEEG.

There are several reasons for implementing these tasks in Step 1. In some TMS-compatible EEG systems, data is collected at a high sampling rate (up to 20 kHz) to capture the wide bandwidth of the large-amplitude TMS pulse. Combined with a dense array of EEG electrodes, file sizes can become extremely large. To reduce file size without significantly affecting data integrity, downsampling is performed with *pop_resample* from the EEGLAB toolbox. This function ensures an anti-aliasing (low-pass) filter is applied before resampling to a lower rate, thereby reducing the effects of aliasing. In general, users are encouraged to verify the degree and impact of downsampling on their respective dataset. If necessary, users may also choose to skip downsampling at this stage and apply it after removal of large-amplitude TMS artifact. In addition to downsampling, channel coordinates must be specified to ensure proper functionality of many visual features of TMSEEG. Epoching is necessary to extract and analyze time-locked responses to TMS. Finally, baseline correction or demeaning can be applied to reduce the effect of low frequency drifts or other low-frequency artifacts. Users can specify the time period used for this correction in the Settings menu.

#### Step 2. Removing the Large-Amplitude TMS Artifact

This step involves the removal of the short-lived, large-amplitude TMS artifact (generally <10 ms following each pulse). As outlined earlier, the TMS pulse artifact is present in almost every trial and cannot be simply removed by trial deletion as in the case of sporadic artifacts. The presence of this artifact impacts the functionality of the subsequent processing steps and thus it is best to remove early in the data processing pipeline. In TMSEEG, users can remove the data segment containing the artifact from each trial and then concatenate the remaining data segments. This removal does not significantly alter data integrity (see “Removal of TMS Decay Artifact in ICA Round 1” in Validation Section for a demonstration) but improves the attenuation of the decay artifact in a subsequent step of the workflow.

**Figure 4:**
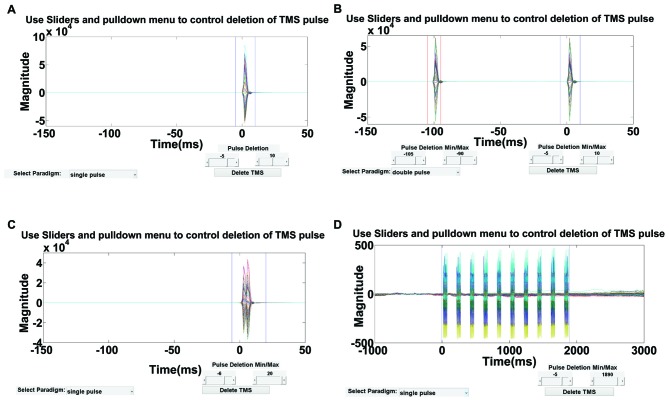
**Step 2 GUI for removing the large-amplitude TMS pulse artifact.** Figures display the average butterfly plot of all EEG data epochs plotted over a time range of −150 ms to 50 ms relative to time of the test pulse in **(A–C)**, and −1000 ms to 3000 ms relative to the first repetitive TMS (rTMS) pulse in **(D)**. *X*-axes represent time in milliseconds and *y*-axes represent the amplitude of the EEG signal in μV. Sliders indicate the time range for data removal. The user can specify the time range for deletion using the provided sliders for four types of TMS-EEG paradigms:** (A)** single-pulse, **(B)** long-interval (100 ms) paired-pulse, **(C)** short-interval (5 ms) paired-pulse, and **(D)** rTMS (specifically intermittent theta burst stimulation).

As depicted in Figure 4, an interactive visualization is provided in this step so that users can verify the time range of deletion. This time range should ideally include the large-amplitude TMS pulse artifact but not the TMS decay artifact, which will be removed in subsequent steps. For multi-pulse paradigms, similar steps can be used to delete the artifact after each pulse. Using Step 2 GUI (Figure 4), the user has the flexibility to define the TMS paradigm and interactively select the time range(s) for removal based on their paradigm. By deleting the contaminated data segment(s), each epoch is shortened by the artifact period and the remaining data segments are concatenated. For visualization, an empty buffer space can be used in place of the deleted time periods in Step 10.

#### Step 3. Removing Bad Trials and Channels (Round 1)

EEG recordings are highly susceptible to sporadic artifacts that may render entire trials unusable. Removal of trials with sporadic noise will facilitate detection of the exponential decay artifact in round 1 of ICA (Steps 4–5) and other general artifacts in round 2 of ICA (Steps 7–8). To facilitate this process, Step 3 integrates several novel features to provide an intuitive interface for users to display, tag, and delete trials, channels, or trials within a single channel. Although it is important to identify and remove data segments contaminated by noise, it is also important to retain as much of the data as possible. In general, removing an excessive number of channels may affect the performance of ICA. To find *N* stable ICA components using X channels (where *N* ≤ X) ICA requires on order of N^2^ data points from each channel to derive N^2^ ICA weights for the unmixing matrix (Delorme et al., 2007). Ideally, this number should be as large as possible; thus, data retention is an important prerequisite for successful decomposition.

To summarize, there are four main novel features integrated in Step 3 to facilitate the visualization of multidimensional TMS-EEG datasets. First, data segments with sporadic artifacts can be easily tagged or untagged for deletion through simple mouse-clicks. Second, to better discriminate segments with artifacts, Step 3 supports several measures of noise estimation that can be selected by the user. Third, the contribution of noise to TEP is clearly illustrated through the concurrent visualization of the TEP with and without the tagged data segment. Finally, for quality control, a record of the deleted channels and trials is automatically saved for later revision if necessary.

**Figure 5:**
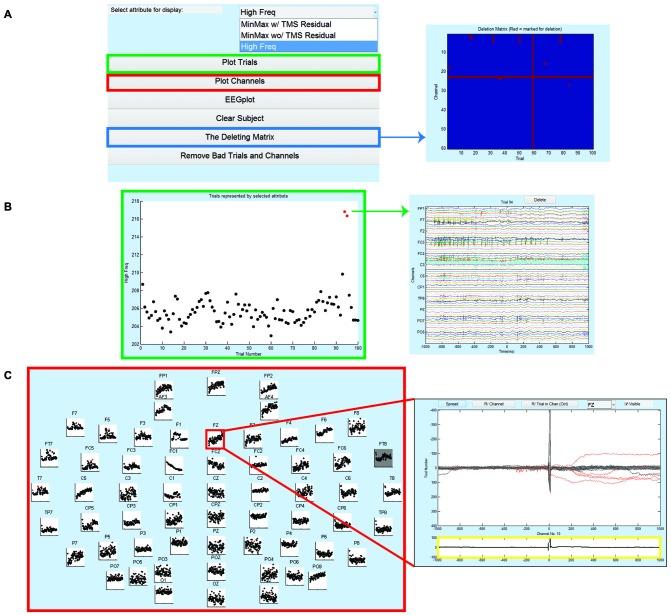
**Step 3 GUI for data cleaning. (A)** The GUI for Step 3 is shown on the left. Right panel depicts the CHAN_DELETION matrix displaying the trials and channels tagged for removal. This matrix provides a visual inspection of deleted data for quality control (*x*-axis is the trial number and *y*-axis is the channel number). In the main GUI, the user can select an ATTRIBUTE value for data display using the drop-down menu. **(B)** GUI for Plot Trials, displaying trials by their average ATTRIBUTE value (left); *x*-axis is the trial number and *y*-axis is the ATTRIBUTE value. Selecting a single dot opens the corresponding trial data (right), and in this window the user can choose to delete the entire trial (dot becomes red) or selectively remove specific channels in the trial. In the right window, *x*-axis represents time in milliseconds relative to presentation of TMS pulse and *y*-axis is the channel labels. **(C)** GUI for Plot Channels, displaying trials by their average ATTRIBUTE value for every channel (left). Selecting a channel’s scatter plot opens the corresponding channel data (right) and in this window the user can choose to delete the entire channel (scatter plot turns dark gray) or selectively remove specific trials within the channels (trials are marked in red and corresponding dot in scatter plot turns red). In the right window, *x*-axis represents time in milliseconds relative to presentation of TMS pulse and *y*-axis represents the amplitude of the EEG signal in μV. The bottom plot in the right window (highlighted in yellow) is the mean TEP (averaged across trials) for the selected channel.

TMSEEG supports several measures of noise estimation for artifact quantification. These measures can be selected by the user through the ATTRIBUTE menu in the Step 3 Main GUI (Figure 5A). The current choices for noise quantification are based on frequency and amplitude characteristics of the signals. Users also have the option of selecting the time window(s) used for noise estimation. This option allows users to be selective about inclusion or exclusion of time points immediately around the TMS pulse(s) in noise estimation. The current list of ATTRIBUTE measures can be expanded by the user to include additional features. After selecting an appropriate ATTRIBUTE value, users can analyze and remove trials and channels using two different methods of visualization:

1. **Deletion by trial**: Accessed through the “Plot Trials” GUI, each trial (shown as a single data point on the scatter plot) is represented by its ATTRIBUTE value averaged across all channels (Figure 5B). Clicking on a data point in the Plot Trial window displays the corresponding trial (i.e., an individual epoch) over all channels (Figure 5B). Within this window, the user can either delete the entire trial or selectively delete up to C noisy channels within the trial, where C is a user-defined threshold specified in the setting tab. If less than C channels within a trial are deleted, these C channels are interpolated for the duration of the trial at the completion of this step. Deleting more than C channels within the trial will delete the trial. In the “Validation” Section of this article, the impact of varying the threshold level of selective channel interpolation is illustrated.
2. **Deletion by channel**: In the “Plot Channels” GUI, each channel has an associated scatter plot illustrating the value of the chosen ATTRIBUTE (*y*-axis) for every trial of the channel (*x*-axis; Figure 5C). Clicking on a channel’s scatter plot brings up an analysis GUI (Figure 5C) allowing the deletion of an entire channel or the deletion of up to *T* trials within the channel. Deleting less than *T* trials within a channel will result in the interpolation of the channel in those trials. Deleting more than *T* trials within a channel will delete the channel across all trials. If a channel is deleted across all trials, the channel can be interpolated after ICA in Step 10. In addition, users can visualize any given trial over all channels and delete the entire trial if necessary.

The data can also be visualized and tagged for artifacts using the standard EEGLAB display for EEG data (i.e., using *eegplot* function). This functionality is available directly through the Step 3 Main GUI. At the completion of Step 3, TMSEEG maintains a record of Trial/Channel pairings marked for deletion in a standalone CHAN_DELETION file. Users also have the option to visualize time segments marked for deletion through the Step 3 Main GUI before removing the marked data (Figure 5A).

#### Steps 4–5. Removing the TMS Decay Artifact Using ICA (Round 1)

After the large-amplitude pulse artifact is removed, ICA can be used to identify the exponential decay artifact. This artifact lasts roughly 40 ms from the onset of a TMS pulse and cannot be removed by deletion without significant data loss. Removal of decay artifact by ICA therefore allows for later processing steps to proceed unhindered by this artifact. This is shown in the “Validation” Section. ICA can also be used to isolate other artifactual components through a second round of application as explained under Steps 7–8. Calling ICA twice at different stages allows the intermediate use of filters and potentially enhances the performance of the second application of ICA.

As in previous steps, TMSEEG provides an interactive display for the ICA component removal process by displaying the effect of removing a selected component on averaged TEPs in real-time. The process of removing the TMS decay artifact is executed through two steps, Steps 4 and 5. Step 4 runs ICA decomposition with a user-defined number of components. By default, the pop_runica function from EEGLAB is used with the FASTICA algorithm (Hyvärinen, 1999). However, users could specify an alternative ICA algorithm through the Settings menu. Step 5, then provides visualization of the *N* largest ICA components displayed in order of decreasing variance, where *N* is a user-defined setting. Due to the large amplitude of a decay artifact, corresponding ICA component(s) can be identified by the first few largest ICA components.

**Figure 6:**
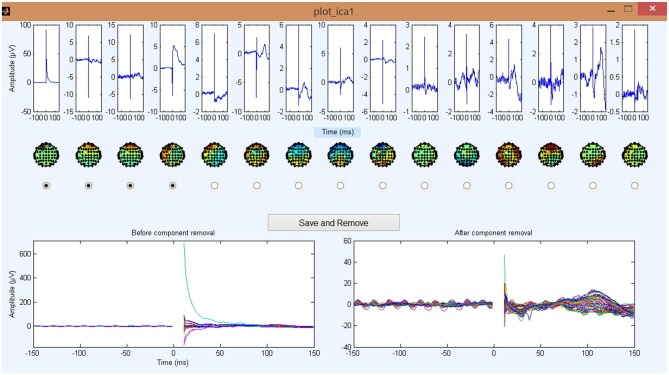
**Step 5 GUI for removing the TMS decay artifact.** The plots in the top row are the 15 largest independent component analysis (ICA) components averaged across trials and sorted by component variance. Corresponding scalp plots are shown below. The bottom left butterfly plot displays the EEG data averaged over all trials before the removal of any ICA components (each colored line presents a channel). The bottom right butterfly plot shows the updated EEG data averaged over all trials after the deletion of tagged ICA components. Note the drastic change in the amplitude of the *y*-axis from the left panel (1000 μV) to the right panel (100 μV) and the unmasking of smaller evoked signals. For all waveform plots, *x*-axis represents time in milliseconds relative to presentation of TMS pulse and *y*-axis represents the amplitude of the EEG signal in μV.

The Main GUI for Step 5 is illustrated in Figure 6. The top panel displays averaged ICA component plots in the time-domain along with corresponding topographical maps. The bottom-left panel of the GUI displays a butterfly plot of the original input data, while the bottom-right panel updates to reflect the data after removing the user-selected ICA components. This is an important and unique feature implemented in TMSEEG for visual verification. It assists the user in identifying the minimum number of ICA components for removal to recover the underlying TEPs. It should be noted that removing components at this stage can decrease the number of components available for the second round of ICA.

#### Step 6. Filtering

After the decay artifact is removed, filtering is applied to exclude data outside the expected frequency range of brain activity. This includes the removal of power line noise, high frequency noise from muscle artifacts and low frequency noise such as drifts and perspiration artifact. Users can choose between a finite impulse response (FIR) and an infinite impulse response (IIR) filter. A notch filter is also available for the removal of power line noise (50 Hz or 60 Hz). This step is implemented after the removal of the large-amplitude TMS artifact to prevent distortion of data by filters in the presence of TMS artifacts (Virtanen et al., 1999).

#### Steps 7–8. Removing Residual TMS and General EEG Artifacts Using ICA (Round 2)

At this stage, the majority of artifacts have been removed from the data. Steps 7 and 8 are implemented to remove other artifacts that can be detected by ICA decomposition including: (i) remaining TMS pulse or decay artifacts; (ii) ocular (EOG) artifacts; (iii) muscle (EMG) artifacts; (iv) cardiac signals (EKG); (v) issues with electrode contact or impedance; (vi) baseline drift; and finally (vii) AEPs.

Similar to ICA analysis in Steps 4–5, this process is divided into two steps. In Step 7, ICA is applied and the data file containing the ICA weights is saved. In Step 8, components are reviewed and tagged for removal. This division allows the user to easily experiment with various configurations of ICA component removal without the need to re-run ICA. More specifically, Step 7 runs ICA using the* pop_runica* from EEGLAB with the FastICA algorithm and a user-selected number of components. Step 8 then uses the derived ICA matrix to project all ICA components and their corresponding topographical maps. A persistent COMP_DELETION file is also generated to store the ICA components tagged for removal in Step 8. This allows the quick re-loading of tagged artifact components for further revisions if necessary.

**Figure 7:**
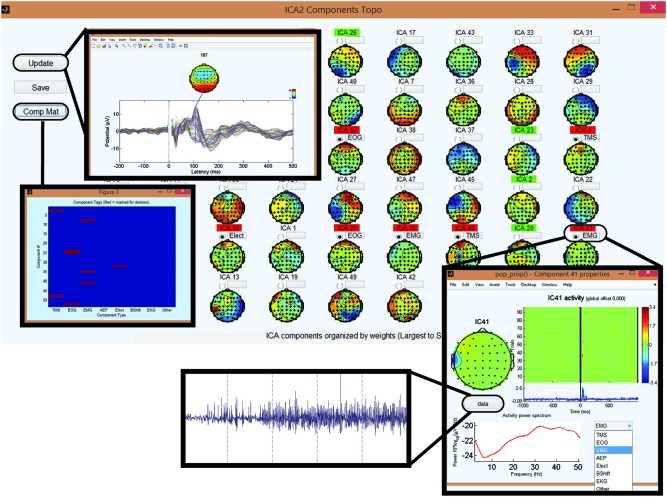
**Step 8 GUI for removing artifactual components in second round of ICA.** Topographical display of the ICA components derived in the second round of ICA in order of variance (largest to smallest). Selecting the gray button above a component will display the temporal, spectral, and spatial characteristics of the corresponding component. Within this “child” GUI, selecting the data button will display the time domain characteristics of the ICA component over each trial. The user can identify the type of artifact using the drop-down menu. This marks the component for deletion. Viewing a component will highlight the component name in green, and marking it for deletion will highlight it in red. Update button on the top left of the main GUI displays the averaged butterfly plot of the EEG data assuming the deletion of the marked components. Comp Mat button on the top left of the main GUI is used to visualize the components marked for deletion for quality control (*x*-axis is the component type and *y*-axis is the component number).

The interface for Step 8 (Figure 7) includes two novel and interactive features to guide users through the process of ICA-based artifact rejection. First, it allows the user to analyze the components, and using a simple drop-down menu, tag components for removal based on their amplitude, duration, frequency and spatial information. The second novel feature is a visual quality control check through the illustration of the averaged TEP butterfly plot before and after the removal of selected ICA components.

#### Step 9. Removing Bad Trials and Channels (Round 2)

Step 9 allows the user to remove any remaining noise across channels and trials before channel interpolation and subsequent statistical analysis. This step creates a second CHAN_DELETION file to keep a record of deleted channels and trials. GUI features available in Step 9 are identical to the features available in Step 3.

#### Step 10. Final Processing

Depending on the type of analysis intended for a dataset, the user may choose to apply a few final processing steps. In Step 10, TMSEEG performs three tasks in the following order: (i) interpolation of channels deleted in Steps 3 and 9; (ii) re-referencing of channel data (optional); and (iii) the addition of a buffer space for time period(s) deleted in Step 2 (optional). Interpolation is performed using the* pop_interp* function from EEGLAB. By default, the function is specified to use the spherical method, which assumes spherical head geometry to estimate scalp potentials at the deleted electrode location. Re-referencing can be performed with any number of EEG electrodes based on user specification. Lastly, the user can specify in the settings tab whether an empty buffer space should be added for visualization purposes. This step concludes the processing of EEG data from raw TMS-EEG signals to clean TEPs. At any given step during the workflow, users can visualize the progressive removal of artifacts through the averaged TEP butterfly plot by clicking on the “View Step” button in the Main GUI (Figure 2), or continue data processing in EEGLAB. Finally, all the settings specified through the TMSEEG workflow (Steps 1–10) are saved in a MATLAB data file providing comprehensive documentation of the data processing methodology.

### Software Architecture and Implementation

**Figure 8:**
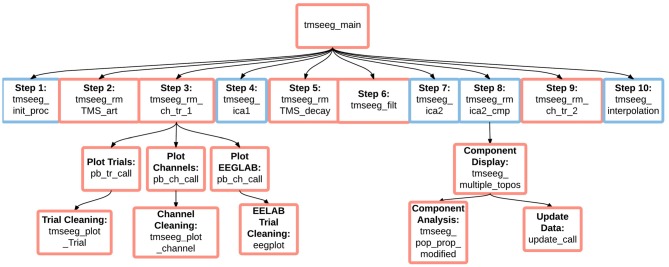
**TMSEEG software architecture.** An illustration of the basic dependencies and relationships between TMSEEG functions. Arrows indicate that a higher level function calls on a lower level “child” function. Blue boxes indicate a functional step, while red boxes indicate that the step spawns a child GUI.

TMSEEG is an open-source toolbox written in MATLAB and distributed through the GNU General Public License. Figure 8 illustrates the program hierarchy and main functions called by TMSEEG. To provide a simple and flexible program structure, the toolbox relies on EEGLAB functions to avoid redundancy between processing functions. This allows us to provide a compact tool with less than 5000 lines of code. In general, the software architecture of TMSEEG is designed towards modularity, i.e., ease of refining/adding to the processing workflow. Users can modify the existing framework to incorporate TMS-EEG paradigms beyond the currently supported protocols and integrate customized algorithms for TMS-EEG signal processing.

Each step in TMSEEG is designed as a self-contained modular process, creating new EEG data structures at the completion of each step to accommodate data interchangeability between processing steps. If information is required by multiple data processing steps, it is saved in separate files for easy loading. For example, trials and channels marked for deletion are saved in CHAN_DELETION files and ICA components marked for deletion are saved in COMP_DELETION files. If the user chooses to reprocess a step, datasets from all subsequent processing steps are automatically deleted.

## Validation

In this section, sample TMS-EEG datasets from five subjects were used to illustrate and validate TMSEEG. All subjects gave their written informed consent and the protocol was approved by the Center for Addiction and Mental Health in accordance with the Declaration of Helsinki. Data examples are extracted from EEG datasets recorded during active and sham single-pulse, active paired-pulse, and active cortical silent period paradigms. Resting motor threshold was defined as the minimum stimulus intensity that elicited an MEP of >50 μV in 5 of 10 trials (Rossini et al., 1994) from the right abductor pollicis brevis muscle. Stimulation intensity for single- and paired-pulse paradigms were determined such that a mean peak-to-peak MEP of 1 mV amplitude was elicited over 20 trials. Sham was administered at the same intensity as active stimulation but with the coil angled at 90° from the scalp resting on one wing of the coil. Intensity for the cortical silent period paradigm was at 140% of resting motor threshold. These suprathreshold monophasic TMS pulses (Magstim200, Magstim Company Ltd., UK) were applied every 10 s in cortical silent period paradigm and every 5 s in the rest of paradigms. In active stimulation, the TMS coil was angled 45° from the midline to the left primary motor cortex and the left dorsolateral prefrontal cortex (DLPFC). The left DLPFC was localized with MRI based neuronavigation (Brainsight, Rogue Research, Montréal, QC, Canada) at MNI co-ordinates *x* = −35, *y* = 45 and *z* = 38. Each paradigm presented up to 100 TMS pulses (i.e., trials). EEG was concurrently recorded with 64-channel EEG (Neuroscan Synamps2) system at a 20 kHz sampling rate. The reference electrode was placed on the vertex between CZ and CPZ electrodes. During initial processing, data was downsampled to 1000 Hz and channels with poor contact were removed. Two-second EEG epochs (−1000 ms to +1000 ms) were extracted from each TMS trial and baseline corrected (−650 ms to −250 ms).

Four features of TMSEEG will be validated in the following sections. These include: (i) selective interpolation of channels; (ii) removal of large-amplitude TMS artifact to extract the TMS decay artifact; (iii) removal of artifacts using ICA; and (iv) performance of TMSEEG with various TMS-EEG paradigms.

### Selective Data Interpolation

Selective interpolation is the interpolation of channel(s) within selected trial(s) as opposed to across all trials. This feature can be used to avoid the deletion of entire channels or trials that contain infrequent instances of sporadic artifacts. Implemented in Steps 3 and 9, selective interpolation is applied when the number of trials tagged for deletion in a single channel is below the user-defined threshold value. In TMSEEG, the default threshold setting is 5% of total number of channels or trials. This default setting implies that when more than 5% of total trials in a channel are tagged for removal, the entire channel is deleted. Similarly, when more than 5% of total channels in a trial are tagged for removal, the entire trial is deleted. The following sections evaluate the integrity of interpolated channels as a function of threshold values. Selective interpolation is demonstrated for Step 3 of the workflow.

#### Interpolation of a Channel Within a Trial

**Figure 9:**
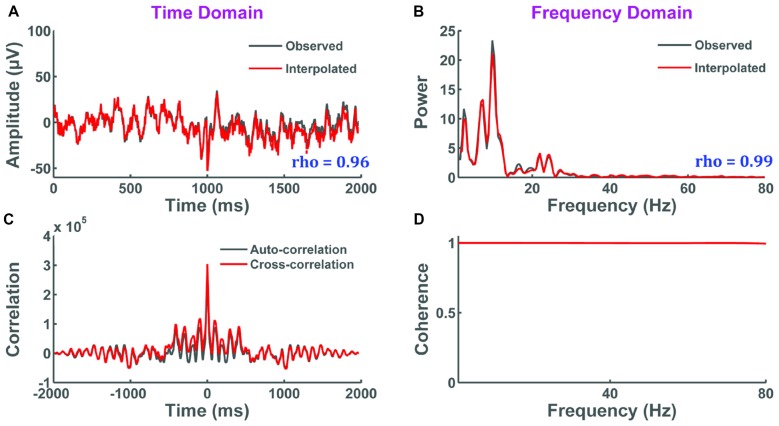
**The impact of selective interpolation on data integrity.** In this figure, we compare data from channel FC3 before (original) and after (interpolated) selective interpolation of a trial within FC3. **(A)** Amplitude of the original signal overlapped with the amplitude of the interpolated signal showing high correlation between the two signals in the time domain. *X*-axis represents time in milliseconds and *y*-axis represents the amplitude of the EEG signal in μV. **(B)** Power spectral density of the original signal overlapped with the power spectral density of the interpolated signal showing high correlation between the two signals in the frequency domain. *X*-axis represents frequency in Hertz and *y*-axis represents the spectral power of the EEG signal in μV^2^/Hz. **(C)** The cross-correlation between the original and interpolated signal closely matches with the autocorrelation of the original signal. *X*-axis represents the various time lags in milliseconds and *y*-axis represents the correlation coefficients at every time lag. **(D)** The coherence plot shows perfect coherence indicating that there is a high degree of linear dependency between the interpolated signal and the original signal. *X*-axis represents frequency in Hertz and *y*-axis represents the magnitude-squared coherence estimates using Welch’s averaged periodogram method at each frequency.

In this test, selective interpolation of a channel within a single trial is evaluated. The original and interpolated signals are compared through: (i) correlation of their amplitude in the time domain; (ii) correlation of their power spectrum magnitude in the frequency domain; (iii) cross-correlation in the time domain; and (iv) coherence in the frequency domain. Results show that all evaluation measures indicate close approximation of the original signal by interpolation (Figure 9). Correlation was high in the time (*r* = 0.96, *p* < 0.001) and frequency domain (*r* = 0.99, *p* < 0.001), the cross-correlation function visually matched the autocorrelation function of the original signal, and near perfect coherence was seen over the selected bandwidth of the EEG signal. Collectively, these results demonstrate that the interpolation of a single trial within a channel has minimal effect on data integrity.

#### Interpolation of a Channel Across *T* Trials

**Figure 10:**
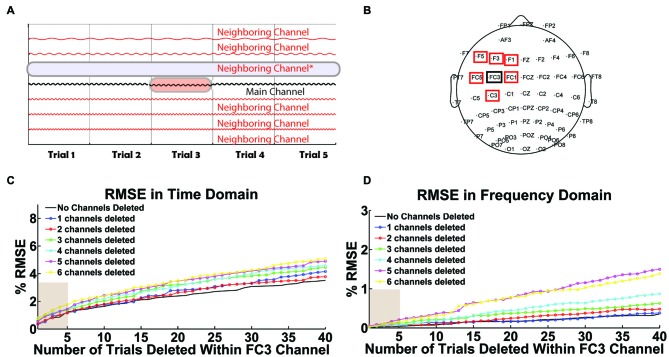
**User-defined threshold for selective interpolation. (A)** The schematic illustrates the data array modification when a user tags a trial within a channel (highlighted in red) or a full channel (highlighted in blue) for removal in Steps 3 or 9. Tagged trials within a channel are interpolated while tagged channels are deleted.** (B)** The scalp map highlights the main channel selected for interpolation (FC3 in black) and its neighboring channels that could be deleted. In **(C,D)**, *x*-axes represent the total number of trials interpolated within channel FC3 and *y*-axes represent the corresponding percent root mean square error (% RMSE). Legend is used to illustrate the total number of deleted neighboring channels. These two plots depict the error in data interpolation in the **(C)** time domain and **(D)** frequency domain. They illustrate a linear increase in interpolation error when an increasing number of trials are interpolated within FC3. Interpolation error also increases with the cumulative deletion of neighboring channels.

The second test illustrates the performance of selective interpolation when *T* trials of channel X are interpolated and *N* channels surrounding channel X are also deleted (see Figure 10A for visualization). For this test, we set *T* to range from 1 to 40 trials and *N* to range from 1 to 6 neighboring channels. To validate the correlation between original and interpolated signals, trials and channels with minimal noise were selected. Therefore, channel X was chosen to satisfy the following requirements: (i) it is close to the stimulation site; (ii) the channel’s data is relatively clean; and (iii) it has at least six neighboring channels that also have relatively clean data. Tests were performed with channel FC3 from a TMS-EEG dataset collected during single-pulse left DLPFC stimulation at rest. The performance of selective interpolation was monitored using percentage root mean square error (% RMSE) in the time and frequency domain. A simple schematic diagram is used to visualize the data matrix when a single trial is removed within the main channel (FC3) given a neighboring channel is also deleted (Figure 10A). The cumulative deletion of up to six neighboring channels of FC3 was implemented in the following order: FC1, F3, C3, FC5, F5 and F1 (Figure 10B). For example, in Figures 10C,D, two deleted channels imply the deletion of channels FC1 and F3, three deleted channels imply the deletion of channels FC1, F3 and C3, etc.

Figures 10C,D show the impact of selective interpolation as a function of the user-defined threshold value (*T*) and the number of deleted neighboring channels (*N*). As hypothesized, results indicate that the interpolation error increases with increasing values of *T* and *N* in both the time (Figure 10C) and frequency domain (Figure 10D). The default value of *T* = 5 (out of 100 trials) can be seen as a reasonable threshold for selective interpolation within trials. The user has the flexibility to alter this threshold value as needed.

### Removal of TMS Decay Artifact in ICA Round 1

The TMSEEG toolbox combines two processing steps to remove the largest TMS-induced artifacts in EEG data. The first is to delete the short data segment containing the large-amplitude TMS pulse artifact (in Step 2) and the second is to use ICA to extract the component(s) associated with the TMS decay artifact (Step 4–5). As mentioned previously, the order of these two procedures is essential for the proper detection of an exponential decay artifact. We will illustrate this using two scenarios.

**Figure 11:**
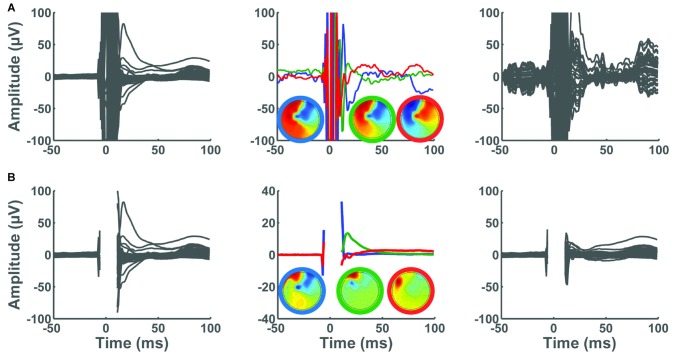
**Consequence of removing the large-amplitude TMS pulse artifact on the performance of ICA.** In all panels, x-axes represent time in milliseconds relative to presentation of TMS pulse and y-axes represent the amplitude of the EEG signal in μV. The left panels show the average butterfly plot of the TEPs, depicting mean TEP (averaged across trials) for each channel before removing ICA components. The center panels depict the three largest ICA components identified in Step 4. In these panels, the three topographical maps are color coded to match their corresponding ICA components in the time domain. The right panels illustrate the average TEP after removing the three ICA components. **(A)** TMS pulse artifact was not deleted in Step 2. The ICA was not able to remove the decay artifact. **(B)** TMS pulse artifact was cut by removing a data segment between −5 ms to 10 ms. The TMS decay components were successfully removed using ICA.

Consider a scenario where the user chooses to bypass Step 2 and retain all data points, and a second scenario in which a short data segment containing the large-amplitude TMS pulse artifact (−5 ms to +10 ms) is marked for deletion. Figure 11 illustrates the ability of ICA to derive components associated with the TMS decay artifact in each of these scenarios. In the first scenario, preserving the data segment containing the large-amplitude TMS pulse artifact limits the performance of ICA in detecting the TMS decay artifact (Figure 11A). In this scenario, the data remain contaminated with substantial TMS-related artifacts. In the second scenario, we observe that deleting the time segment containing the large-amplitude TMS pulse artifact enables isolating and subsequently removing the TMS decay artifact with a small number of ICA components. By removing the three largest ICA components containing the decay artifact, much of the TMS decay artifact was successfully removed (Figure 11B).

### Removal of Artifacts Using ICA Round 2

**Figure 12:**
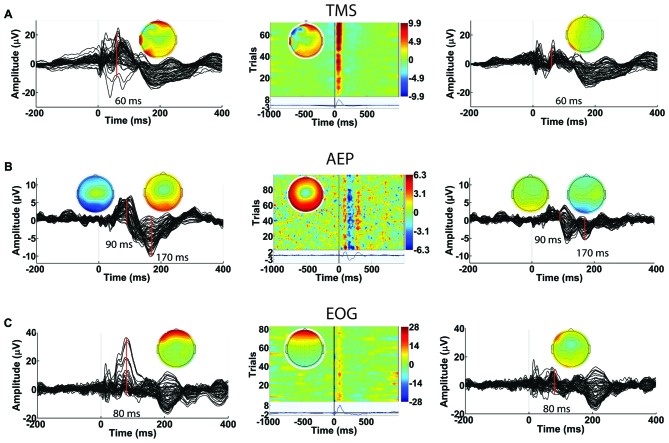
**Examples of artifacts that can be identified in Round 2 of ICA in Step 8.** In each row, the left panel depicts the average butterfly plot of the TEPs before removing the artifact (*x*-axis represents time in ms relative to presentation of TMS pulse and *y*-axis represents the amplitude of the EEG signal in μV). The center panel displays the ICA component associated with the artifact (*x*-axis represents time in milliseconds relative to presentation of TMS pulse, *y*-axis represents the trial number and a color scale is used to depict the amplitude of the ICA component in μV). The right panel shows the averaged butterfly plot of the TEPs after removing the artifact (*x*-axis represents time in milliseconds relative to presentation of TMS pulse and *y*-axis represents the amplitude of the EEG signal in μV). Topographical maps in each panel illustrate the scalp projection of the artifactual component at the latency of the artifact. Rows illustrate the impact of removing **(A)** a residual TMS artifact with latency of 60 ms (identified in data collected during suprathreshold single pulse TMS applied to the left dorsolateral prefrontal cortex [DLPFC]), **(B)** auditory evoked potential (AEP) with latency of 90 ms and 170 ms (identified in data collected during suprathreshold single pulse sham TMS applied to the left motor cortex), and** (C)** eye blink artifact with latency of 80 ms (identified in data collected during suprathreshold single pulse TMS applied to the left DLPFC).

**Figure 13:**
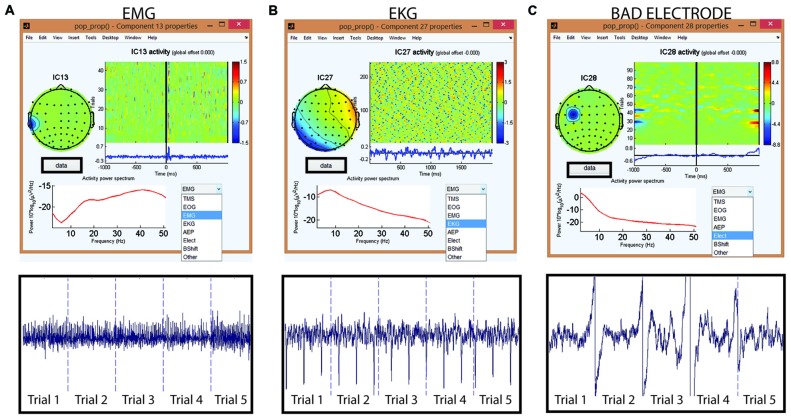
**Tagging artifactual ICA components through TMSEEG toolbox.** The top row displays the TMSEEG GUI for classification of ICA components and the visualization of the spatial, temporal and spectral properties of three ICA components **(A–C)**. In each window, the spatial characteristics of the component is displayed on the top left corner (topographical plot), the magnitude of the component across trials is displayed in the top right corner (*x*-axis represents time in milliseconds relative to TMS pulse and *y*-axis represents trials), and the frequency spectrum of the component is displayed in the bottom left corner (*x*-axis represents frequency in Hertz and *y*-axis represents the power spectral density). The drop-down menu on the bottom-right allows for a convenient classification of the artifact type. The second row depicts the ICA components in the time-domain over five continuous trials. The user can access this time-domain depiction of components through the “Data” button embedded in the GUI. The displayed artifacts are: **(A)** muscle artifact (electromyography [EMG]) in the left panel, **(B)** cardiac artifact (electrocardiographic [EKG]) in the middle panel, and **(C)** bad electrode artifact in the right panel.

The second round of ICA targets the identification and removal of residual TMS-induced artifacts and general EEG artifacts (Makeig et al., 1996; Delorme and Makeig, 2004; Joyce et al., 2004; Delorme et al., 2007). With a comprehensive GUI in Step 8, users have access to several key component characteristics (e.g., time, frequency and spatial characteristics) to accurately label components for removal. Additionally, the user can visualize the effects of removing selected ICA components on the averaged TEP waveform. Together, these features help users through the process of identifying and tagging physiological artifact components such as eye blinks, eye movement, muscle artifacts and AEPs, and non-physiological artifact components such as baseline drifting, electrode movements and any residual TMS artifacts. The characteristics of artifactual components specific to TMS-EEG datasets (amplitude, duration, and spatial distribution) have been described in detail in a recent article (Rogasch et al., 2014). Figure 12 shows the capability of TMSEEG in extracting TMS, AEP and EOG artifacts. Figure 13 illustrates the characteristics of other smaller amplitude artifacts as detected through ICA such as muscle, cardiac and bad electrode noise.

### Performance With Various TMS-EEG Paradigms

**Figure 14:**
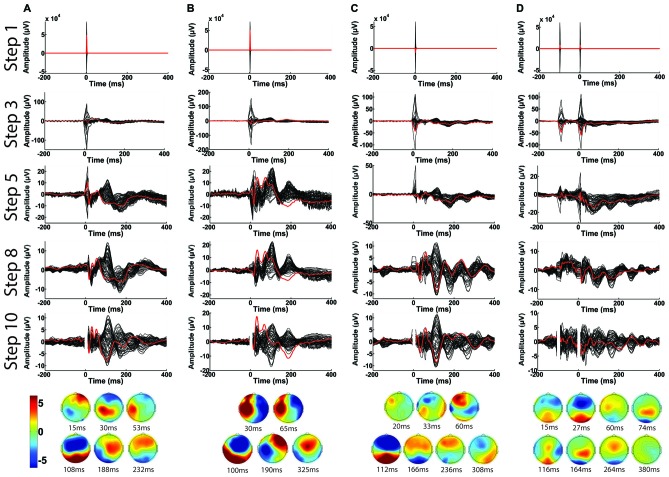
**Illustration of the capability of TMSEEG in processing several types of single-pulse and paired-pulse TMS paradigms.** Panels illustrate the average butterfly plot of the TEPs at the completion of each major processing step (1, 3, 5, 8 and 10). The channel closest to the stimulation site is highlighted in red in each plot (C3 for left motor cortex and F3 for DLPFC). *X*-axes represent time in milliseconds relative to presentation of TMS pulse and *y*-axes represent the amplitude of the EEG signal in μV. The topographical plots at the bottom illustrate the voltage scalp map at each significant peak of the butterfly plot from Step 10 in μV. The displayed TMS-EEG paradigms are:** (A)** single-pulse stimulation applied to the left motor cortex at rest, **(B)** single-pulse stimulation applied to the left motor cortex during active muscle contraction (cortical silent period paradigm), **(C)** single-pulse stimulation applied to the left DLPFC at rest, and** (D)** paired-pulse (long-interval cortical inhibition paradigm) applied to the left DLPFC at rest.

In Figure 14, we demonstrate the overall capability of the TMSEEG toolbox. The progressive refinement of averaged TEP waveforms (from Step 1 to Step 10) is illustrated using four different TMS-EEG paradigms: single-pulse stimulation applied to the left motor cortex at rest (Figure 14A) and during active muscle contraction (i.e., cortical silent period paradigm; Figure 14B), single-pulse stimulation applied to the left DLPFC (Figure 14C), and paired-pulse stimulation (i.e., long interval cortical inhibition) applied to the left DLPFC (Figure 14D). Across all paradigms, the large-amplitude TMS artifact was removed by cutting EEG segments of −5 ms to +10 ms around each TMS pulse in Step 2. IIR bandpass (1–80 Hz) and notch filters (60 Hz) were applied in Step 6. In Step 10, the deleted channels were interpolated, data was re-referenced to an average reference, and the time segment removed in Step 2 was replaced with a buffer space. As illustrated, TMSEEG can successfully recover the TEPs for each paradigm, comparable to previous literature (Komssi and Kähkönen, 2006; Lioumis et al., 2009; Farzan et al., 2013; Rogasch et al., 2014).

## Discussion

In this article, we have introduced TMSEEG, a novel, streamlined GUI-based tool for processing TMS-EEG data. First, a description of the step-by-step procedure was provided to illustrate the functionality of TMSEEG toolbox. Then, the technical capabilities of the toolbox were highlighted through validation of its functionality. Finally, the toolbox was used to illustrate processing of TMS-EEG data in various single- and paired-pulse paradigms, thereby confirming its utility in streamlined TMS-EEG data processing.

### User-Level Features

The TMSEEG interface is designed to provide a simple display of the TMS-EEG data processing workflow. Through the main GUI panel, users can easily track the order of the processing steps and specify the configuration parameters for each step. To enhance the artifact rejection process, step-specific GUIs provide the user with a comprehensive set of information on the artifacts. For example, within steps 3, 5, 8 and 9, the user can view multidimensional EEG data in the time domain (over trials or channels), frequency domain, or spatial domain to enable fast detection and removal of various types of artifacts. Finally, TMSEEG provides multiple online visual feedbacks to illustrate the consequence of artifact removal on EEG signals. This allows the user to monitor the progress and quality of data processing throughout the workflow.

### Performance-Level Features

In the “Validation” Section, a number of performance-level features of TMSEEG were illustrated and validated. These include selective interpolation of channels within selected trials to help with data retention, removal of the large-amplitude TMS artifact to assist in the detection of the decay artifact, as well as the removal of other TMS-related and general EEG artifacts such as EOG, EMG, EKG, AEP, and bad electrode through ICA. The structure of the TMSEEG workflow also ensures that data modification performed at any given step systematically leads to subsequent processing steps. Finally, TMSEEG provides a comprehensive environment that is flexible to accommodate the processing of various single- or multi-pulse TMS-EEG paradigms.

### Comparison With Existing Tools

TMSEEG is a stand-alone GUI and the first to offer a comprehensive, streamlined workflow for processing TMS-EEG data. The toolbox is built upon the commonly-used EEG data processing toolbox, EEGLAB. Fieldtrip is another MATLAB-based toolbox for EEG, magnetoencephalography (MEG), and TMS-EEG data processing. However, the proper use of functions within Fieldtrip depends on the expertise and programming skills of the user. Compared to these toolboxes, one novel aspect of TMSEEG is its simplified user interface. Due to the high dimensionality of EEG data, a comprehensive visualization of multiple TMS-EEG specific signal attributes is essential during the artifact rejection process. Moreover, the unique implementation of numerous visual inspections comparing raw and cleaned data, throughout the pipeline, promotes quality control. While experienced users may be able to implement such visualizations in Fieldtrip, or through scripting in EEGLAB, both novice and experienced users will benefit from the visualization features provided in the TMSEEG interface. Specifically, our design allows for efficiency, ease-of-use, and standardization of TMS-EEG data processing. Additionally, the simple and intuitive interface of TMSEEG currently accommodates single-pulse, paired-pulse and basic rTMS EEG analysis. It can also be easily extended to other TMS protocols. Currently, features specific to data retention vary between Fieldtrip and TMSEEG. In Fieldtrip, users can choose to remove artifacts by dividing single trials into short time segments and interpolating the segments deemed artifactual across channels. TMSEEG, however, promotes data retention by providing users with the added option to delete selective trials from individual channels. Another feature unique to TMSEEG is the automatic saving of data processing information (such as bad trials, channels, and components) in a matrix. This matrix can then be easily visualized, reviewed, modified by re-processing the data, and also compared across different files and studies for data monitoring.

### Importance of Standardized Methodology for TMS-EEG Data Processing

A major impediment to the widespread adoption of TMS-EEG is the lack of a standardized data processing methodology. TMS-EEG outcomes may vary with variations in data processing parameters, differences in the data processing methodology, or subjectivity from the lack of a systematic approach in artifact selection. By introducing an organized workflow which follows systematic practices of data analysis, our aim is to optimize TMS-EEG data cleaning and to assess and promote data compatibility, test-retest reliability and inter-rater reliability across studies. For example, through the use of validated algorithms, and the flexibility to incorporate new algorithms developed for TMS-EEG processing, the TMSEEG toolbox can be adapted to extract most types of artifacts from TMS-EEG data. The implementation of a standard procedure along with a modularized architecture can promote data replication. In addition, detailed documentation of the parameters used in the data processing routine (e.g., all user-input settings are saved in a MATLAB data file) and deleted artifacts (also saved in MATLAB data files) may help identify the sources of variability between users and between studies. Finally, the intuitive interface of TMSEEG is designed to improve the accessibility of TMS-EEG data processing for users with different levels of expertise.

### Plans for Future Releases

We are continuously improving the flexibility and features of TMSEEG. One of the main reasons for implementing TMSEEG in a MATLAB environment is to provide compatibility with existing MATLAB toolboxes for EEG analysis. This includes EEGLAB (Delorme and Makeig, 2004), Brainstorm (Tadel et al., 2011), and Fieldtrip (Oostenveld et al., 2010). Future versions of TMSEEG will aim to implement easy accessibility to functions of these toolboxes while maintaining the convenience and flexibility provided by a user interface. Overall, the aim of TMSEEG is the simplification of data inspection, artifact rejection, multimodal analysis, source localization and statistical analysis in a comprehensive, efficient and reliable software suite.

TMSEEG is designed towards flexibility and adaptability to accommodate multiple neurophysiological experiments. For example, a user can modify or rearrange the workflow to accommodate for alternate methods of data processing. Furthermore, the workflow can be expanded to accommodate different experimental conditions including multi-pulse (more than two pulses) and various rTMS paradigms. Future versions of the software will include GUI-based functions to facilitate the modification and customization of TMSEEG.

TMSEEG also aims to provide increased flexibility for algorithm experimentation. For example, the current version of TMSEEG relies largely on the FastICA algorithm for artifact rejection. However, future versions will aim to introduce two additional features to improve artifact rejection in TMSEEG. The first is to provide users with the option to choose from different algorithms and apply the algorithm of choice for each artifact. For example, it has been suggested that different algorithms are suited for the removal of different sources of artifacts (e.g., see Lyzhko et al., 2015). With this added functionality, users can implement a series of customized steps (e.g., such as Step 2) for optimized removal of artifacts specific to a recording system or the site of stimulation (e.g., see Mäki and Ilmoniemi, 2011). A second feature for a future version is the automatic tagging of various artifacts (e.g., see Chaumon et al., 2015) through the use of a database or library of artifacts. A database can support the application of machine-learning algorithms to automatically classify and reject artifacts based on a user’s approach. This novel extension could improve the speed and reliability of artifact detection with TMSEEG.

## Summary

TMSEEG is the first open-source GUI-based MATLAB application for TMS-EEG data processing. It enables the removal of TMS-induced and general EEG artifacts through several functionalities such as artifact quantification and intuitive data visualization. In addition, through a simple GUI, TMSEEG provides extensive support for novice users while providing flexibility through ease-of-modification of its features for experienced users. In general, the toolbox is designed to streamline the process of multidimensional TMS-EEG data processing and promote the widespread utility and standardization of TMS-EEG methodology for adaptation in various disciplines of neuroscience. We invite interested readers to refer to http://www.tmseeg.com/downloads/ to download a copy of the toolbox.

## Author Contributions

SA, MF, LGD, YM, FF contributed to the software design. All authors contributed to the improvement of the tool. SA, MF, FF wrote the manuscript. All authors edited and approved the manuscript for publishing. All authors agree to be accountable for all aspects of the work.

## Conflict of Interest Statement

The authors declare that the research was conducted in the absence of any commercial or financial relationships that could be construed as a potential conflict of interest.
